# New and Useful Veterinary Instruments

**Published:** 1895-07

**Authors:** James A. Waugh


### NEW AND USEFUL VETERINARY INSTRUMENTS.

REVIEWED BY DR. JAMES A. WAUGH.

Dr. L. L. Conkey, V.S., of Grand Rapids, Michigan, has invented a new and very useful trocar and canula tracheotomy tube which is a wonderful improvement and far superior to all similar instruments. It consists of a semi-oval trocar eight and one-half inches long, with a diamond-shaped head one and one-half inches long by one-half wide and five-sixteenths thick, surmounting a shoulder fitting the canula. The canula is six and three-quarter inches long, one-half by one-quarter inch in diameter, and has an oblong opening centrally in the broadest oval sides, one and one-half by one-half inch wide, to admit the air into the trachea. The instrument is held in position by two set screws fitting into two neatly capped washers that fit over the ends of the canula containing four holes in each end. It must be seen and used before being understood and appreciated by modern veterinarians, who will invariably praise it as a practical instrument. I have used it successfully after repeated failures and unfavorable results with the other styles of tracheotomy tubes, and this also has been the experience of my brother, William J. Waugh, V. S., Third Calvary, U. S. A.

Prof. Hughes, M.R.C.V.S., of Chicago Veterinary College, has invented an equine stomach trocar and canula for puncturing the horse's stomach in case of acute indigestion, with gaseous distention, especially where the gases fail to pass into the bowels. This trocar and canula is similar to the average British or French instruments, except that the canula is eleven and one-half inches long. It should always be used on the right side, about the same region as generally selected for the ordinary operation. I have not yet had an opportunity to use this instrument, but will report results in the future. I was chagrined lately at losing a valuable and fast trotting stallion, besides three draught-horses, which suffered with acute indigestion complicated with accumulation of gas in the stomach. I tapped one horse sixteen times and struck a pocket of gas every time. I tapped a fine and valuable hearse horse six times without success, and the seventh proved successful and removed large quantities of gases and saved the animal's life. I always use antiseptics and clip the hair off a spot when I use a lance to cut the skin before using the trocar and canula, and by these means prevent the formation of abscesses.

